# S1PR1 regulates ovarian cancer cell senescence through the PDK1-LATS1/2-YAP pathway

**DOI:** 10.1038/s41388-023-02853-w

**Published:** 2023-10-12

**Authors:** Yi-Ping Tao, Heng-Yan Zhu, Qian-Yuan Shi, Cai-Xia Wang, Yu-Xin Hua, Han-Yin Hu, Qi-Yin Zhou, Zi-Lu Zhou, Ying Sun, Xiao-Min Wang, Yu Wang, Ya-Ling Zhang, Yan-Jun Guo, Zi-Ying Wang, Xuan Che, Chun-Wei Xu, Xian-Chao Zhang, Michal Heger, Su-Ping Tao, Xin Zheng, Ying Xu, Lei Ao, Ai-Jun Liu, Sheng-Bing Liu, Shu-Qun Cheng, Wei-Wei Pan

**Affiliations:** 1 Department of Cell Biology, College of Medicine, Jiaxing University, 118 Jiahang Road, Jiaxing, 314001 China; 2 Zhejiang Chinese Medicine University and Jiaxing University Master Degree Cultivation Base, Jiaxing University, 118 Jiahang Road, Jiaxing, 314001 China; 3 Department of Anesthesiology, Jiaxing Maternity and Child Health Care Hospital, Affiliated Women and Children Hospital, Jiaxing University, Jiaxing, Zhejiang Province 314001 China; 4 Institute of Basic Medicine and Cancer (IBMC), Chinese Academy of Sciences, No. 1 Banshan East Street, Gongshu District, Hangzhou, 310022 China; 5 Institute of Information Network and Artificial Intelligence, Jiaxing University, 118 Jiahang Road, Jiaxing, 314001 China; 6 Jiaxing Key Laboratory for Photonanomedicine and Experimental Therapeutics, Department of Pharmaceutics, College of Medicine, Jiaxing University, 118 Jiahang Road, Jiaxing, 314001 China; 7 Department of Pharmaceutics, Utrecht Institute for Pharmaceutical Sciences, Utrecht University, Universiteitsweg 99, 3584 CG Utrecht, the Netherlands; 8 Laboratory of Experimental Oncology, Department of Pathology, Erasmus MC, Dr. Molewaterplein 40, 3015 GD Rotterdam, the Netherlands; 9 Department of Gynecology and Obstetrics, Affiliated Hospital of Jiaxing University, Jiaxing, 314000 China; 10 Department of Pathology, the 7th Medical Center, General Hospital of PLA, Beijing, 100700 China; 11 Department of Hepatic Surgery VI, Eastern Hepatobiliary Surgery Hospital, Second Military Medical University, 225 Changhai Road, Shanghai, 200438 China; 12 G60 STI Valley Industry & Innovation Institute, Jiaxing University, 118 Jiahang Road, Jiaxing, 314001 China

**Keywords:** Senescence, Cancer genomics

## Abstract

Cell senescence deters the activation of various oncogenes. Induction of senescence is, therefore, a potentially effective strategy to interfere with vital processes in tumor cells. Sphingosine-1-phosphate receptor 1 (S1PR1) has been implicated in various cancer types, including ovarian cancer. The mechanism by which S1PR1 regulates ovarian cancer cell senescence is currently elusive. In this study, we demonstrate that S1PR1 was highly expressed in human ovarian cancer tissues and cell lines. S1PR1 deletion inhibited the proliferation and migration of ovarian cancer cells. S1PR1 deletion promoted ovarian cancer cell senescence and sensitized ovarian cancer cells to cisplatin chemotherapy. Exposure of ovarian cancer cells to sphingosine-1-phosphate (S1P) increased the expression of 3-phosphatidylinositol-dependent protein kinase 1 (PDK1), decreased the expression of large tumor suppressor 1/2 (LATS1/2), and induced phosphorylation of Yes-associated protein (p-YAP). Opposite results were obtained in S1PR1 knockout cells following pharmacological inhibition. After silencing LATS1/2 in S1PR1-deficient ovarian cancer cells, senescence was suppressed and S1PR1 expression was increased concomitantly with YAP expression. Transcriptional regulation of S1PR1 by YAP was confirmed by chromatin immunoprecipitation. Accordingly, the S1PR1-PDK1-LATS1/2-YAP pathway regulates ovarian cancer cell senescence and does so through a YAP-mediated feedback loop. S1PR1 constitutes a druggable target for the induction of senescence in ovarian cancer cells. Pharmacological intervention in the S1PR1-PDK1-LATS1/2-YAP signaling axis may augment the efficacy of standard chemotherapy.

## Introduction

Ovarian cancer is a common gynecological malignancy associated with high mortality rates [1]. Early detection is complex and treatment is complicated by chemotherapy resistance [2]. It is therefore imperative to identify early markers for optimal screening of ovarian cancer as well as novel, more effective therapeutic avenues [3]. Cell senescence is a biological process with multiple functions, including tumor suppression [4], which plays an important role in the biological processes of ovarian cancer. The occurrence, development, and treatment of ovarian cancer are closely related to senescence. Senescence further plays a role in the response to chemotherapy and biotherapy of ovarian cancer [5]. However, the mechanisms that underlie senescence in ovarian cancer cells are currently elusive.

Sphingosine-1-phosphate (S1P) regulates cell differentiation, proliferation, and migration, among other physiological processes [6]. Previous studies have demonstrated that S1P signaling is critical in the pathogenesis of cancer [7, 8]. S1P is produced by the phosphorylation of sphingosine kinase 1/2 (SPHK1/2) on the cell membrane, which binds to S1P receptors (S1PRs). S1PR isoforms include S1PR1-5, where S1PR1 plays an important role in carcinogenesis [9, 10]. The S1PR1 signaling pathway mediates the production of various oncogenic regulators involved in tumor angiogenesis and immune cell migration [11–13]. In ovarian cancer, the S1P/S1PR1/3 pathway is involved in regulating angiogenesis [14]. Whether S1PR1 regulates senescence in ovarian cancer cells is presently unknown. Furthermore, S1PR1 can mediate the Yes-associated protein (YAP) signaling pathway in human granulosa cells [15]. However, YAP-S1PR1 cross-talk and the role of YAP in regulating cellular senescence in ovarian cancer remains unclear.

We therefore investigated S1PR1 expression patterns in human ovarian cancer and the role of S1PR1 in cell cycle regulation, proliferation, and senescence in cultured cells and a xenograft mouse model. Knocking down S1PR1 or activating S1PR1 with S1P revealed that S1PR1 inhibits senescence by stimulating PDK1 expression and inhibiting LATS1/2 and YAP expression. The high expression of S1PR1 in ovarian cancer tissues and cells was further linked to chemotherapy resistance. The decrease in S1PR1 expression in ovarian cancer cells promoted senescence and enhanced chemotherapeutic efficacy of cisplatin. Mechanistic studies showed that YAP stimulates S1PR1 expression, thus constituting a positive feedback loop. This feedback regulation of S1PR1-PDK1-LATS1/2-YAP may be involved in the regulation of ovarian cancer cell senescence. Our findings give rise to potential intervention strategies for ovarian cancer, which can be explored as standalone treatments or as adjuvant therapies to improve the efficacy of standard chemotherapeutic drugs.

## Results

### S1PR1 is highly expressed in human ovarian cancer tissues and cells

**Fig. 1:**
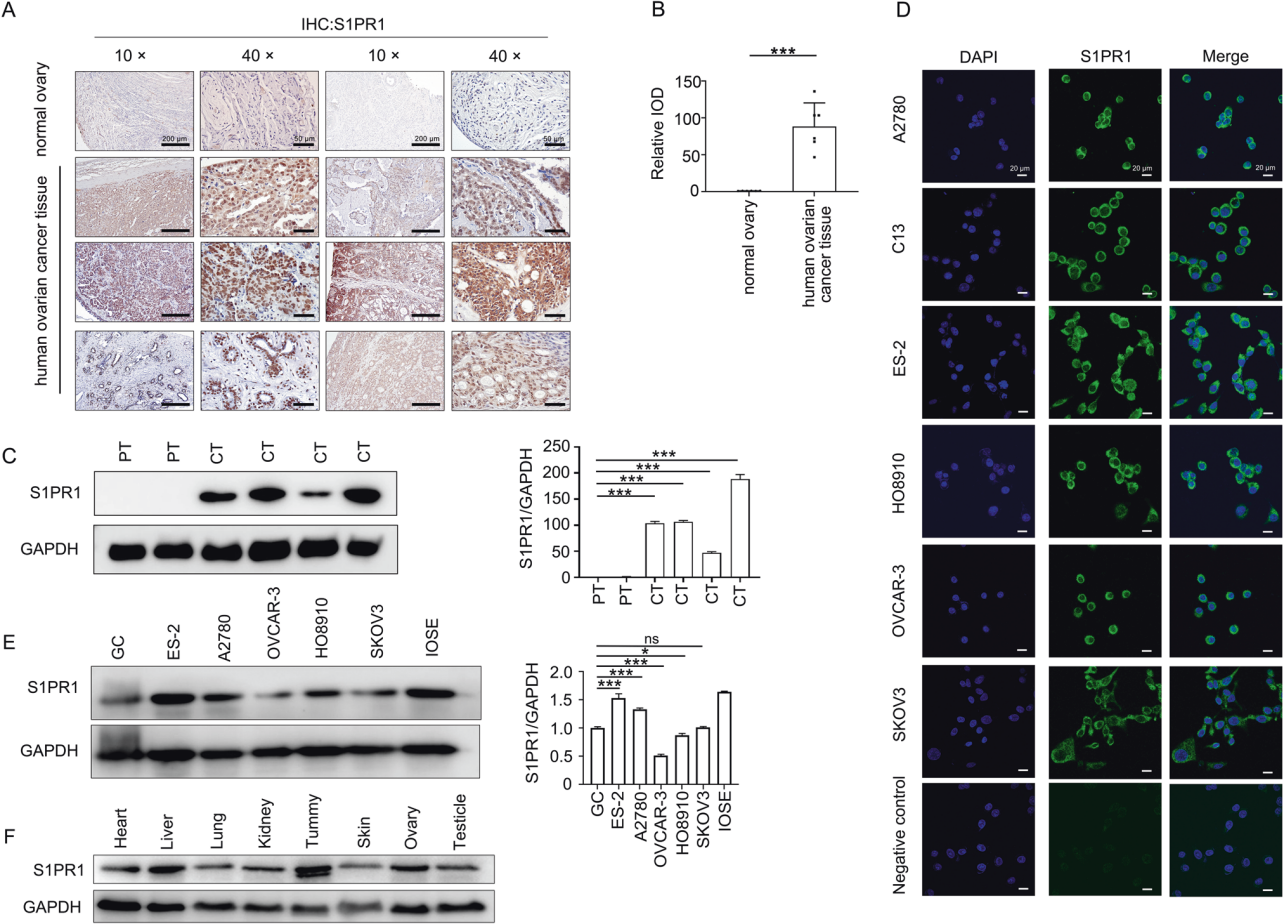
Expression of S1PR1 in ovarian cancer tissues and paracancerous tissues, various organs, and ovarian cancer cell lines. **A** Immunohistochemical analysis of S1PR1 in human ovarian cancer tissues (*n* = 6) and adjacent tissues (*n* = 2). *Scale bar, 200* *µm* (10 ×); *Scale bar, 50* *µm* (40 ×). **B** Quantitative analysis of immunohistochemical results. Student’s *t*-test; ***p < 0.001. **C** Western blot analysis of S1PR1 in human ovarian cancer tissues (CT) (*n* = 4) and paracancerous tissues (PT) (*n* = 2; left) and relative quantitative analysis (right). One-way ANOVA; ***p < 0.001. GAPDH was used as control. **D** Immunofluorescence analysis of S1PR1 in ovarian cancer cells. DAPI was used to counterstain nuclei (blue). *Scale bar, 20* *µm*. **E** Western blot analysis of S1PR1 in GC (granular cells derived from normal ovaries), ovarian cancer cell lines (ES-2, A2780, OVCAR-3, HO8910, SKOV3), and IOSE (ovarian cancer epithelial cell line; left) and relative quantitative analysis (right). GAPDH was used as control. One-way ANOVA; ns, *p* > 0.05; *p < 0.05; ***p < 0.001. **F** Western blot analysis of S1PR1 in organs (liver, lung, heart, kidney, tummy, skin, ovary, and testicle). GAPDH was used as control.

We observed that S1PR1 was highly expressed in human ovarian cancer tissues (*n* = 6), but relatively low in normal ovarian tissues (*n* = 2) (Fig. 1A, B). Western blotting showed that S1PR1 was highly expressed in ovarian cancer tissues (*n* = 4), but mildly expressed in paracancerous tissues (*n* = 2) (Fig. 1C). Immunofluorescence staining showed that S1PR1 protein was localized on the cell membrane of SKOV3, OVCAR-3, HO8910, ES-2, A2780, and C13 ovarian cancer cells (Fig. 1D). Western blotting revealed that S1PR1 was highly expressed in ES-2 and A2780 cells, but weakly expressed in granular cells derived from normal ovaries and OVCAR-3 cells (Fig. 1E). In normal organs, S1PR1 was highly expressed in the tummy, liver, kidney, and ovarian tissues (Fig. 1F). Collectively, S1PR1 is overexpressed in ovarian cancer tissues and cells compared to non-cancerous controls and may therefore play a role in ovarian cancer biology.

### S1PR1 deletion inhibits ovarian cancer cell proliferation

**Fig. 2:**
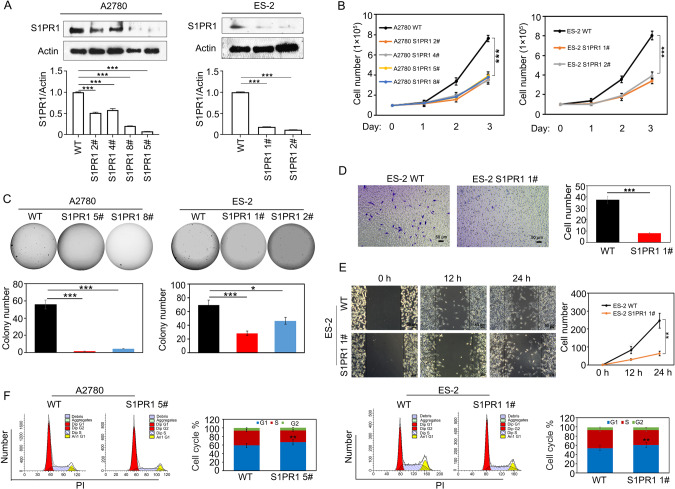
S1PR1 knockout affects the proliferation, migration, and the cell cycle of ovarian cancer cells. **A** Western blot analysis of S1PR1 expression in wild-type and S1PR1 knockout ovarian cancer cells (A2780 and ES-2). One-way ANOVA; ***p < 0.001. β-actin was used as control. **B** Cell counting assay was used to detect the proliferation ability of wild-type ovarian cells and S1PR1 knockout cells (A2780 and ES-2). Counting was performed on days 1, 2, and 3 after inoculation and entailed 3 technical replicates per sample. One-way ANOVA; ***p < 0.001. **C** Soft agar cloning assay was used to compare the proliferation ability of wild-type ovarian cancer cells and S1PR1 knockout cells. Values represent mean ± SD of three independent experiments. One-way ANOVA; *p < 0.05, ***p < 0.001. **D** Transwell assay analysis of the migration ability of S1PR1 knockout cells (ES-2). *Scale bar, 50* *µm*. Values represent mean ± SD of three independent experiments. Student’s *t*-test; ***p < 0.001. **E** Scratch test analysis of the migration ability of S1PR1 knockout cells (ES-2). *Scale bar, 50* *µm*. Values represent mean ± SD of three independent experiments. Student’s *t*-test; **p < 0.01. **F** Flow cytometry was employed to analyze the changes in the cell cycle in S1PR1 knockout A2780 and ES-2 cells. Data (each cell line experiment was repeated in triplicate) are presented as mean ± SD.

The S1P/S1PR signaling pathway is involved in regulating the invasive potential of ovarian cancer [16]. To investigate whether S1PR1 affects the proliferation of ovarian cancer cells, S1PR1 was knocked out in A2780 and ES-2 ovarian cancer cells using CRISPR/Cas9 [17]. Western blotting was performed to detect knockdown effects. S1PR1 expression was partially reduced in ovarian cancer cells (Fig. 2A). Cell counting for 3 days and soft agar clone formation assays showed that S1PR1 deletion inhibited the proliferation and clone formation (Fig. 2B, C). Next, we found that S1PR1 deletion inhibited cell migration in ES-2 cells using scratch and transwell assays (Fig. 2D, E). In addition, S1PR1 deletion affected the cell cycle of ES-2 and A2780 cells, which were arrested in the G1 phase (Fig. 2F). These results indicate that S1PR1 plays a vital role in ovarian cancer cell proliferation.

### S1PR1 affects ovarian cancer cell senescence through the PDK1-LATS1/2-YAP pathway

**Fig. 3:**
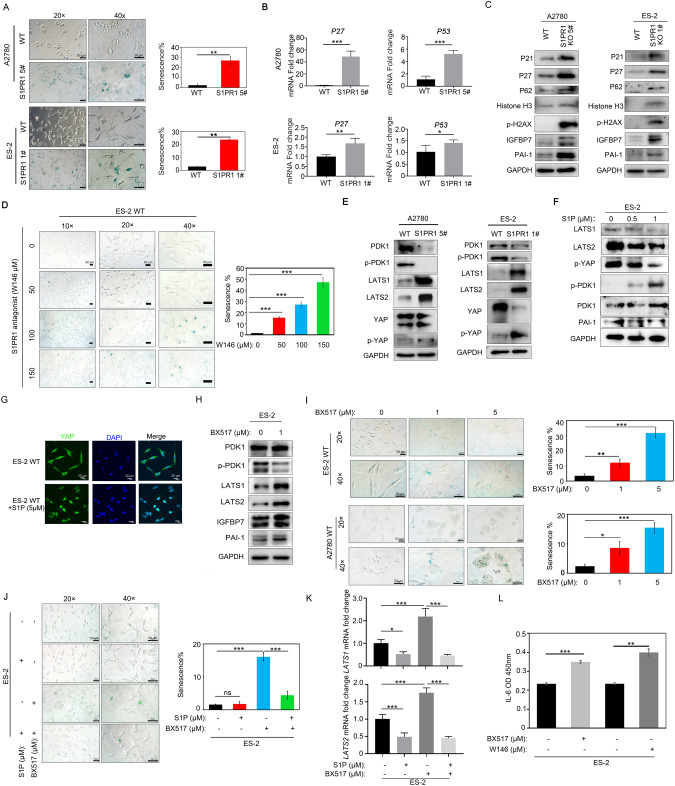
S1PR1 regulates ovarian cancer cell senescence through the PDK1-LATS1/2-YAP pathway. **A** β-galactosidase staining was used to qualitatively (left) and quantitatively (right) analyze senescence changes in S1PR1 knockout A2780 and ES-2 cells. *Scale bar, 50* *µm*. Values represent mean ± SD of three independent experiments. Student’s *t*-test; **p < 0.01. **B** q-PCR analysis of *P27* and *P53* expression in wild-type and S1PR1 knockout ovarian cancer cells. Values represent mean ± SD of three independent experiments. Student’s *t*-test; *p < 0.05, **p < 0.01, ***p < 0.001. **C** Western blot showing the expression of P21, P27, P62, IGFBP7, PAI-1, histone H3, and p-H2AX in wild-type cells and S1PR1 knockout cells. GAPDH was used as control. **D** β-galactosidase staining was used to qualitatively (left) and quantitatively (right) analyze senescence changes in ES-2 cells after treatment with S1P1 receptor antagonist W146. *Scale bar, 50* *µm*. Values represent mean ± SD of three independent experiments. One-way ANOVA; ***p < 0.001. **E** Western blot showing expression levels of PDK1, p-PDK1, LATS1, LATS2, YAP, and p-YAP in wild-type ovarian cancer cells and S1PR1-deficient ovarian cancer cells. GAPDH was used as control. **F** Western blot showing expression levels of PDK1, p-PDK1, LATS1, LATS2, PAI-1, and p-YAP after S1P treatment in ES-2 cells. GAPDH was used as control. **G** Immunofluorescence analysis of YAP expression and localization (green) following S1P treatment and no treatment. DAPI was used to counterstain nuclei (blue). *Scale bar, 20* *µm*. **H** Western blot showing the expression of LATS1, LATS2, PDK1, p-PDK1, IGFBP7, and PAI-1 in ES-2 cells after treatment with PDK1 inhibitor BX517. GAPDH was used as control. **I** β-galactosidase staining analysis of senescence changes after BX517 treatment of ovarian cancer cells is shown qualitatively (left) and quantitatively (right). *Scale bar, 50* *µm*. Values represent mean ± SD of three independent experiments. One-way ANOVA; *p < 0.05, **p < 0.01, ***p < 0.001. **J** β-galactosidase staining analysis of WT cell senescence after BX517 (1 µM) and S1P (1 µM) treatment. *Scale bar, 50* *µm*. Values represent mean ± SD of three independent experiments. One-way ANOVA; ns, *p* > 0.05, ***p < 0.001. **K** q-PCR analysis of LATS1/2 after S1P (1 µM) and BX517 (1 µM) treatment of ovarian cancer cells. Values represent mean ± SD of three independent experiments. One-way ANOVA; *p < 0.05, ***p < 0.001. **L** ELISA showing IL-6 levels in ES-2 cells treated with BX517 (2 µM) and W146 (100 µM) separately. Values re*p*resent mean ± SD of three independent experiments. One-way ANOVA; **p < 0.01, ***p < 0.001.

The combined detection of aging biomarkers and cell proliferation can improve the reliability of predicting aging cells in tissue slices [18]. P21, P27, P53, and P62 mediate cell senescence [19–22]. Senescent cells produce a complex secretome (senescence-associated secretory phenotype, SASP) that includes IL-8, IL-6, plasminogen activator inhibitor 1 (PAI-1), and insulin-like growth factor binding protein 7 (IGFBP7), which enhance senescent growth arrest [23]. We observed that S1PR1 deletion promoted cell senescence in ES-2 and A2780 cells (Fig. 3A). Next, we detected the expression of senescence-related genes and proteins in S1PR1knockout ovarian cancer cells. qRT-PCR analysis indicated that senescence genes *P27* and *P53* were increased in S1PR1 knockout ES-2 and A2780 cells (Fig. 3B). Western blotting showed that the expression of aging-related proteins P21, P27, P62, histone-H3, p-H2AX, PAI-1, and IGFBP7 were significantly elevated in S1PR1-deleted cells (Fig. 3C and S1A). After ES-2/A2780 cells were treated with S1P1 receptor antagonist W146 (50 µM, 100 µM and 150 µM), the number of senescent cells increased significantly in a W146 concentration-dependent manner (Fig. 3D and S2A). These results suggest that downregulation of S1PR1 expression or activity promotes ovarian cancer cell senescence.

Recent studies have found that PDK1 co-regulates cell aging [24–27] and that S1PR1 can regulate the expression of key proteins LATS1/2 and YAP in the Hippo pathway [15, 28]. However, whether S1PR1 can regulate aging through PDK1 in ovarian cancer cells and whether LATS1/2 and YAP are involved in this regulation has not been reported. Western blot analysis of S1PR1-deleted cells revealed that the expression of PDK1 was significantly decreased and the expression of LATS1/2 and p-YAP was significantly increased (Fig. 3E and S1C). In addition, q-PCR showed that *LAST1* and *MST1* also increased, and that YAP and YAP downstream target genes *Cyr61* and *Ankrd1* significantly decreased (Fig. S2B). Consequently, S1PR1-PDK1-LATS1/2-YAP pathway may play a role in the regulation of ovarian cancer cell senescence.

To further understand the mechanism of S1PR1 in ovarian cancer cell senescence, we treated ovarian cancer cells with S1P to activate S1PR1 and detected the expression changes of S1PR1-related downstream proteins. Western blotting results show that the expression of PDK1 was increased at 1 µM S1P treatment, while the expression of LATS1/2 and phosphorylation of YAP significantly decreased (Fig. 3F and S1B and S2C). Immunofluorescence also showed that YAP entered the nucleus of ovarian cancer cells after S1P treatment (Fig. 3G). Similarly, PDK1 expression was decreased and LATS1 expression was increased after treatment with S1P1 receptor antagonist W146 at 75 μM (Fig. S2D). These results suggest that S1PR1 may regulate ovarian cancer cell senescence through the PDK1-LATS1/2-YAP pathway.

To verify these results, the PDK1 inhibitor BX517 was used to confirm that PDK1 downregulation promotes ovarian cancer cell senescence. We found that LATS1, LATS2, and the aging-related proteins IGFBP7 and PAI-1 were significantly increased, while p-PDK1 was significantly decreased following treatment with BX517 (1 μM) (Fig. 3H and S1D). Cell senescence assays showed that the number of aging cells was also significantly increased following BX517 treatment at > 1 μM (Fig. 3I). However, S1P treatment inhibited the promoting effect of BX517 on ovarian cancer cell senescence (Fig. 3J). q-PCR results showed that S1P treatment significantly reduced the level of *LATS1/2*, *P21*, and *P53* induced by BX517 (Fig. 3K and S2E), which further proves the promoting effect of S1P on ovarian cancer cell senescence. ELISA results revealed that IL-6 levels were increased significantly after treatment with S1P1 receptor antagonist W146 and BX517 (Fig. 3L). These results suggest that S1PR1 regulates ovarian cancer cell senescence through the PDK1-LATS1/2-YAP pathway.

### Downregulation of S1PR1 induces senescence and increases the sensitivity of ovarian cancer cells to cisplatin (CDDP)

**Fig. 4:**
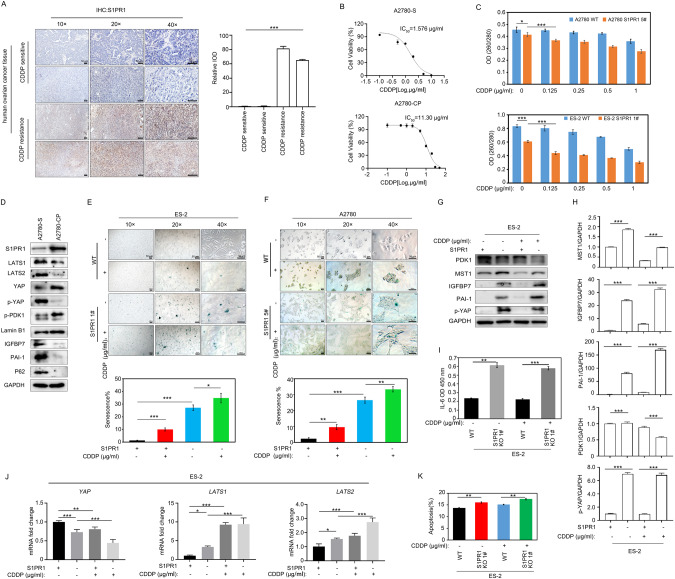
S1PR1 deletion promotes CDDP-induced senescence in ovarian cancer cells. **A** Immunohistochemistry analysis of the expression of S1PR1 in CDDP (cisplatin) chemotherapy-insensitive ovarian cancer tissues (CDDP-resistant) and chemotherapy-sensitive ovarian cancer tissues (CDDP-sensitive; left) and quantitative analysis (right). *Scale bar, 50* *µm*. Values represent mean ± SD of three independent experiments. One-way ANOVA; ***p < 0.001. **B** IC_50_ values of A2780-S (CDDP-sensitive) and A2780-CP (CDDP-resistant) ovarian cancer cells. **C** CCK8 cell proliferation assay analysis of the proliferative propensity after treatment with increasing concentrations of cisplatin. Values represent mean ± SD of three independent experiments. One-way ANOVA; *p < 0.05, ***p < 0.001. **D** Western blot analysis of the expression of S1PR1, LATS1, LATS2, YAP, p-YAP, p-PDK1, lamin B1, P62, IGFBP7, and PAI-1 in A2780-S (CDDP-sensitive) and A2780-CP (CDDP-resistant) ovarian cancer cell lines. GAPDH was used as control. **E**, **F** β-galactosidase assay analysis of the effect of CDDP (0.125 µg/mL) on senescence in wild-type and S1PR1 knockout ovarian cancer cells. *Scale bar, 50* *µm*. Values represent mean ± SD of three independent experiments. One-way ANOVA; *p < 0.05, **p < 0.01, ***p < 0.001. **G**, **H** Western blot analysis of expression levels of PDK1, MST1, IGFBP7, p-YAP, and PAI-1 in wild-type ES-2 cells and S1PR1 knockout ES-2 cells after CDDP treatment (0.125 µg/mL). Values represent mean ± SD of three independent experiments. One-way ANOVA; ***p < 0.001. GAPDH was used as control. **I** ELISA analysis of IL-6 expression in S1PR1 knockout ES-2 cells before and after CDDP treatment (0.125 µg/mL). Values represent mean ± SD of three independent experiments. Student’s *t*-test; **p < 0.01, ***p < 0.001. **J** q-PCR analysis of *LATS1/2, YAP* and *P21* expression in wild-type ES-2 cells and S1PR1 knockout ES-2 cells after CDDP treatment (0.125 µg/mL). Values represent mean ± SD of three independent experiments. One-way ANOVA; *p < 0.05, **p < 0.01, ***p < 0.001. **K** The effect of S1PR1 knockdown on cisplatin-induced apoptosis of ovarian cancer cells was analyzed by flow cytometry. Data (each cell line experiment was repeated in triplicate) are presented as mean ± SD. One-way ANOVA; **p < 0.01.

To investigate whether S1PR1 can affect ovarian cancer chemotherapy resistance through senescence, we first detected its expression in tissues from chemoresistant patients (CDDP-resistant) and chemosensitive patients (CDDP-sensitive). Immunohistochemical analysis evinced that S1PR1 expression was higher in chemoresistant ovarian cancer tissues than in chemosensitive ovarian cancer tissues (Fig. 4A). Next, we verified cisplatin resistance in ovarian cancer cell lines by IC_50_ calculation. The CDDP IC_50_ in A2780-S cells was 1.576 µg/mL while the CDDP IC_50_ in A2780-CP cells was 11.30 µg/mL (Fig. 4B). These results demonstrated that A2780-CP cells are CDDP-resistant and A2780-S cells are CDDP-sensitive, which is consistent with previous studies [29, 30]. Cell proliferation analysis further showed that cisplatin (0.125 µg/mL) significantly reduced the proliferation of S1PR1 knockout cells (Fig. 4C). S1PR1 expression was significantly higher in A2780-CP cells than in A2780-S cells. In addition, the expression of LATS1/2, p-YAP, P62, PAI-1, and IGFBP7 were significantly decreased, while p-PDK1 and lamin B1 were significantly increased in cisplatin-resistant A2780-CP cells (Fig. 4D). This suggests that S1PR1 may play a role in resistance of ovarian cancer cells to chemotherapy. S1PR1 deletion promoted senescence in ovarian cancer cells treated with cisplatin (Fig. 4E, F). When S1PR1 knockout cells were treated with cisplatin, PDK1 protein levels were decreased and MST1 protein levels were increased. S1PR1 deletion promoted cisplatin-induced senescence-related protein IGFBP7 and PAI-1 expression (Fig. 4G, H). Treating S1PR1 knockout cells with cisplatin also increased IL-6 levels (Fig. 4I). q-PCR results further showed that S1PR1 knockdown increased *LATS1/2*, *P21*, and *P53* levels in cisplatin-treated ovarian cancer cells, while *YAP* levels decreased. (Fig. 4J and S2F). In addition, S1PR1 knockdown promoted cisplatin-induced apoptosis of ovarian cancer cells (Fig. 4K). These results indicate that S1PR1 plays a role in the chemotherapy resistance in ovarian cancer.

### S1PR1 deletion suppresses tumor growth and promotes tumor cell senescence in vivo

**Fig. 5:**
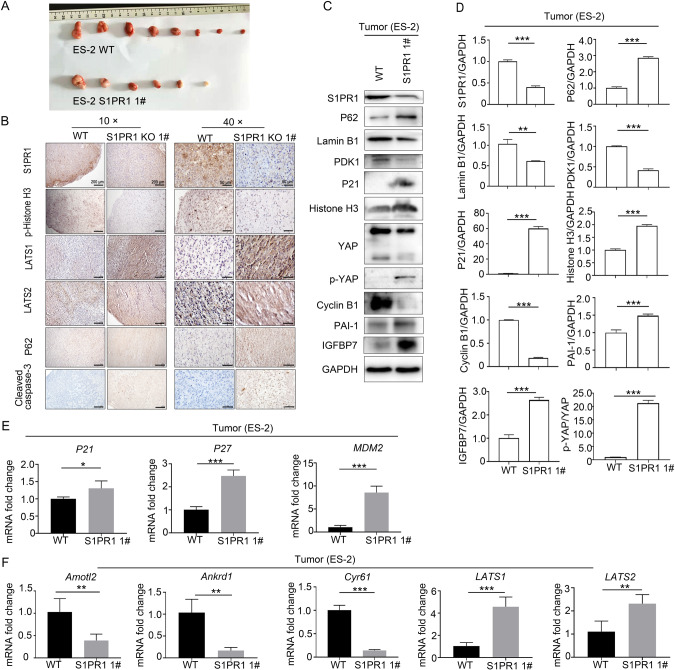
S1PR1 promoted ovarian cancer cell proliferation and inhibited ovarian cancer cell senescence in vivo. **A** Wild-type and S1PR1 knockout ES-2 cells were subcutaneously injected into nude mice (500,000 cells per mouse, *n* = 8 per group). Mice were sacrificed after 20 days. Photographs of resected tumors is shown. **B** Immunohistochemistry staining for S1PR1, LATS1/2, P62, cleaved caspase-3, and p-histone H3 in representative ES-2-derived tumor tissue. *Scale bar, 200* *µm* (10 ×)*. Scale bar, 50* *µm* (40 ×). **C**, **D** Western blot analysis of S1PR1, senescence-associated proteins (P21, P62, histone H3, IGFBP7, PAI-1, and lamin B1), PDK1, p-YAP, cyclin B1, and YAP. GAPDH was used as control. Student’s *t*-test; **p < 0.01, ***p < 0.001. **E** Transcript levels of *P27, P21*, and *MDM2* were analyzed by q-PCR in ES-2-derived tumor tissues. Student’s *t*-test; *p < 0.05, ***p < 0.001. (**F**) *LATS1/2* and YAP downstream genes *Amotl2, Ankrd1*, and *Cyr61* were analyzed by q-PCR in ES-2-derived tumor tissues. Student’s *t*-test; **p < 0.01, ***p < 0.001.

To detect the effects of S1PR1 on ovarian cancer cell proliferation and senescence in vivo, wild-type and S1PR1 knockout ES-2 cells were subcutaneously injected into both flanks of nude mice. At the end of the experiment, the tumor volume in S1PR1 knockout mice was smaller than that in wild-type mice (Fig. 5A). Immunohistochemistry showed that S1PR1 and p-histone H3 (marker of proliferation) protein levels were significantly decreased, whereas LATS1, LATS2, P62, and cleaved caspase-3 protein levels were significantly increased in S1PR1 knockout tumor tissue (Fig. 5B). Western blot results showed that S1PR1, cyclin B1, PDK1, and lamin B1 protein levels were significantly decreased, whereas P21, P62, histone H3, IGFBP7, and PAI-1 protein levels were significantly increased in S1PR1 knockout tumor tissue (Fig. 5C, D). q-PCR results showed that the age-related genes *P27*, *P21*, and *MDM2* were significantly increased in S1PR1-deficient tumors (Fig. 5E). The mRNA levels of the YAP downstream target genes *Amotl2, Ankrd2*, and *Cry61* were significantly decreased, while *LATS1* and *LATS2* mRNA levels were increased (Fig. 5F). These results indicate that S1PR1 deletion inhibited ovarian cancer tumor growth in vivo.

### S1PR1 regulates ovarian cancer cell senescence via positive feedback through the PDK1-LATS1/2-YAP signaling pathway

**Fig. 6:**
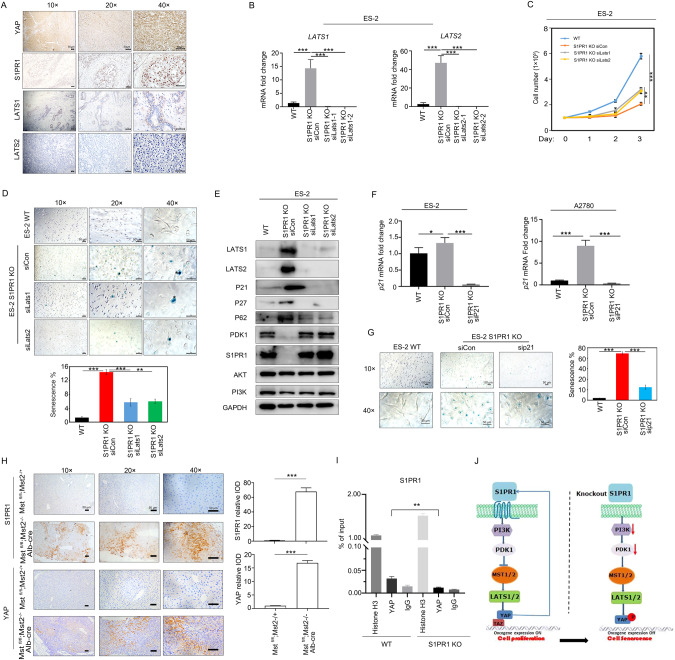
LATS1/2 deletion in S1PR1 knockout cells can rescue cell proliferation and reduce cell senescence. **A** Immunohistochemistry staining for S1PR1, LATS1, LATS2, and YAP in human ovarian cancer tissue. *Scale bar, 50* *µm*. **B** q-PCR for LATS1/2 silencing effect in S1PR1 knockout cells. Values represent mean ± SD of three independent experiments. One-way ANOVA; ***p < 0.001. **C** Cell counting assay results showing the proliferation ability of control cells, S1PR1 knockout cells, and S1PR1 knockout cells with LATS1/2 silencing (A2780 and ES-2). Cells (100,000) were seeded in 6-well plates. Counting was performed on days 1, 2, and 3 after inoculation and entailed 3 technical replicates per sample. One-way ANOVA; **p < 0.01, ***p < 0.001. **D** β-galactosidase staining results showing senescence of wild-type ovarian cancer cells, S1PR1 knockout cells, and S1PR1 knockout cells with LATS1/2 silencing. *Scale bar, 50* *µm*. Values represent mean ± SD of three independent experiments. One-way ANOVA; **p < 0.01, ***p < 0.001. **E** Western blot results for LATS1, LATS2, S1PR1, AKT, PI3K, PDK1, P21, P27, and P62 in wild-type ovarian cancer cells, S1PR1 knockout cells, and S1PR1 knockout cells with LATS1/2 silencing. GAPDH was used as control. **F** q-PCR of transcript levels of *P21* in wild-type cells, S1PR1 knockout cells, and S1PR1 knockout cells with silenced P21. Values represent mean ± SD of three independent experiments. One-way ANOVA; *p < 0.05, ***p < 0.001. **G** β-galactosidase staining was used to analyze senescence changes in S1PR1 knockout cells with P21 silencing qualitatively (left) and quantitatively (right). *Scale bar, 50* *µm*. Values represent mean ± SD of three independent experiments. One-way ANOVA; ***p < 0.001. **H** Immunohistochemistry staining of S1PR1 and YAP in the liver of MST2 knockout mice. *Scale bar, 50* *µm*. Student’s *t*-test; ***p < 0.001. **I** ChIP analysis of the binding of YAP to S1PR1 promoter in ovarian cancer cells. Student’s *t*-test; **p < 0.01. **J** Schematic diagram of the S1PR1 signaling cascade. After the activation of S1PR1 in ovarian cells, the expression of PDK1 increases, which inhibits the expression of MST1/2-LATS1/2 (the key proteins in the Hippo signaling pathway) and increases YAP transcriptional activity. YAP can further promote S1PR1 transcription and tumor cell proliferation. After knocking out or inhibiting the expression of S1PR1, the expression of PI3K and PDK1 decreases, and MST1/2-LATS1/2 is activated, in turn leading to the inactivation of YAP (by phosphorylation) and tumor cell senescence.

Previous studies have found that S1PR1 knockdown or activation can affect the expression of LATS1/2 and YAP (Fig. 3E, F). Next, we determined the expression of SIPR1, YAP, LATS1, and LATS2 in human ovarian cancer tissues using immunohistochemistry. YAP and S1PR1 were highly expressed, and LATS1 and LATS2 were underexpressed (Fig. 6A). To further verify the role of LATS1/2 in ovarian cancer cell senescence, we silenced LATS1 and LATS2 in S1PR1 knockout ovarian cancer cells. qRT-PCR analysis showed that LATS1/2 expression was repressed significantly in S1PR1 knockout cells by siRNA (Fig. 6B). LATS1/2 silencing in S1PR1 knockout cells significantly promoted ovarian cancer cell proliferation (Fig. 6C). Moreover, silencing LATS1/2 in S1PR1 knockout cells significantly decreased ovarian cancer cell senescence as per β-galactosidase staining (Fig. 6D and S2G). Compared to the S1PR1 deletion control, further deletion of LATS1/2 significantly increased PDK1 and S1PR1 expression and decreased P21, P27, and P62 protein expression (Fig. 6E). These data indicate that the S1PR1-PDK1-LATS1/2-YAP positive feedback pathway regulates ovarian cell senescence. However, *LATS1/2* silencing could not completely rescue the senescence phenotype caused by S1PR1 deletion (Fig. 6D). Therefore, we propose that additional genes are involved in senescence regulation by S1PR1. We observed that the senescence phenotype could also be rescued by interfering with the key senescence gene *P21*. To verify the effect of S1PR1 on ovarian cancer cell senescence, *P21* was knocked down in S1PR1 knockout cells (Fig. 6F). The P21-deleted ovarian cancer cells showed a significant decrease in β-galactosidase staining (Fig. 6G and S2H). Collectively, our results suggest that the Hippo pathway is regulated by S1PR1 and contributes to ovarian cancer cell senescence.

In mammals, MST1/2 and LATS1/2 as well as the downstream effectors YAP/TAZ form the core of the Hippo pathway. MST1/2 is involved in the activation of LATS1/2, which inhibits the activity of YAP/TAZ by direct phosphorylation [31]. We obtained liver tissue from MST2 knockout mice, as previous studies demonstrated that deleting MST2 in the mouse liver resulted in rapid liver enlargement and YAP activation [32]. To verify the feedback regulation of S1PR1 by YAP, we examined S1PR1 expression in the livers of MST2 knockout mice. S1PR1 and YAP protein levels were significantly increased in MST2 knockout liver tissue (Fig. 6H), suggesting that YAP activation could induce S1PR1 expression. To further demonstrate the relationship between YAP and S1PR1, a chromatin immunoprecipitation assay was used to detect YAP binding to the S1PR1 promoter. YAP bound to the S1PR1 gene in ovarian cancer cells, while S1PR1 deletion resulted in a significant decrease in YAP binding to the S1PR1 promoter (Fig. 6I). These observations are consistent with a positive role of S1PR1 in YAP activity regulation. These results therefore corroborate the positive feedback regulatory pathway of S1PR1-PDK1-LATS1/2-YAP.

## Discussion

Ovarian cancer is the deadliest gynecological malignancy [33]. Senescence is a common stress response that occurs during the division of DNA-damaged cells. Inducing tumor cell senescence is an effective way to cope with tumorigenesis, which can arrest cell growth and activate anti-tumor immune responses [34, 35]. Our study demonstrated that S1PR1 inhibits ovarian cancer cell senescence. S1PR1 signaling stimulates cancer cell growth and angiogenesis and promotes tumor cell metastasis. S1PRs belong to the GPCR family of heterotrimeric GTP-binding proteins that can transmit intracellular signals [36–40]. S1PR1 is expressed in all ovarian tumors [41]. Moreover, S1PR1 expression is more significant in stages III-IV, high-grade lymph node metastases, and distant metastases of ovarian cancer, and S1PR1/3 is strongly associated with angiogenesis in ovarian cancer [14].

This study revealed that S1PR1 is highly expressed in ovarian cancer tissues and cell lines. Senescent cells are characterized by increased galactosidase activity, cell cycle arrest, increased P16, P21, P27, p19ARF, p53, and PAI-1 expression, lamin B1 loss, and upregulation of growth factors, cytokines, chemokines, and senescence-associated subtilisin proteases [42–44]. We found that S1PR1 deletion resulted in decreased proliferation and migration of ovarian cancer cells and cell cycle arrest in the G1 phase. In addition, levels of IGFBP7, PAI-1, lamin B1, P21, P27, P62, p-H2AX, and other factors related to cell senescence were significantly elevated, indicating that S1PR1 may be involved in regulating ovarian cancer senescence.

S1P activates its cognate receptors, including S1PR1-5, that regulate various physiological and pathological processes such as cardiovascular disease and cancer [45]. S1P induces time-dependent PDK1 activation [46]. PDK1 is involved in the regulation of various cancer signaling pathways and can also be involved in the regulation of aging [24–27]. In the present study, S1P could induce PDK1 expression in ovarian cancer cells. We also found that PDK1 expression decreased significantly, and cell senescence increased in S1PR1-deficient ovarian cancer cells.

However, the pathway through which PDK1 participates in ovarian cancer senescence regulation requires further investigation. Studies have shown that S1P can activate the LATS1/2 and YAP signaling pathways through S1PR1 [15, 28]. YAP is also related to cell senescence, which can alleviate senescence by upregulating FOXD1 expression and prevent astrocyte senescence by CDK6 pathway modulation [47, 48]. In hepatocellular carcinoma, the epidermal growth factor receptor regulates YAP signaling through the PDK1 pathway to promote hepatocellular carcinoma cell growth [49]. Our results showed that, after S1PR1 knockdown in ovarian cancer cells, the expression of LATS1/2 was significantly increased, the level of phosphorylated YAP was increased, and the expression of PDK1 was significantly decreased. Opposite results were obtained after treatment with S1PR1 receptor agonist S1P compared to S1PR1 knockdown (Fig. 3E). S1P treatment or S1PR1 deletion showed that the PDK1-LATS1/2-YAP pathway was involved in ovarian cancer cell senescence, consistent with the in vivo results. The expression of LATS1/2 was significantly increased in S1PR1-deleted ovarian cancer cells. Further silencing of LATS1/2 in S1PR1-deleted cells reduced senescence (Fig. 6D) but did not completely rescue the senescence phenotype caused by S1PR1 deletion. This result suggests that S1PR1 knockout also affects aging phenotypes through other genes or pathways. The P21/P53 signaling pathway is related to senescence [50]. Our study found that S1PR1 deletion increased P21 expression. Corroboratively, silencing P21 also decreased senescence in S1PR1-deleted ovarian cancer cells (Fig. 6F, G).

Recent studies have found that YAP signaling is regulated by S1P receptor activation, and that there is a correlation between YAP and S1P receptor expression levels. The regulation of YAP by S1P was limited to its effect on cell proliferation and did not involve LATS1/2 [51]. The expression of YAP and S1PR1 increased significantly in the liver of MST2 KO mice [32]. Meanwhile, silencing LATS1/2 in S1PR1-deleted cells promoted S1PR1 expression, which indicates that LATS1/2 regulates S1PR1 transcription by inhibiting YAP activity. Furthermore, ChIP experiments also showed a direct relationship between YAP and S1PR1. The LATS1/2-YAP pathway may feed-regulate the expression of S1PR1. Accordingly, we identified a novel S1PR1-PDK1-LATS1/2-YAP positive feedback mechanism that regulates ovarian cancer senescence.

Inducing ovarian cancer cell senescence can reduce chemotherapy doses and the anti-apoptotic ability of tumors [5, 35]. A previous study has shown that the cellular senescence process is an important cause of drug resistance in high-grade serous ovarian cancer (HGSOC) [52]. S1PR1 is associated with CDDP resistance in ovarian cancer [53]. When investigating whether S1PR1 and CDDP resistance are related to senescence regulation in ovarian cancer, we found that S1PR1 expression was enhanced in both cisplatin-resistant ovarian cancer tissues and cisplatin-resistant ovarian cancer cells. Compared to cisplatin-resistant ovarian cancer cells (A2780-CP), the expression of LATS1/2 and p-YAP was increased in cisplatin-sensitive ovarian cancer cells (A2780-S), and the expression of senescence-related proteins was increased (Fig. 4D). S1PR1 knockdown promoted cisplatin-induced apoptosis of ovarian cancer cells (Fig. 4K). S1PR1 depletion inhibited cell proliferation and promoted cell senescence. In bladder and thyroid cancers, S1PR1 antagonist has been found to inhibit tumor proliferation and migration [54]. In pancreatic cancer, FTY720, an S1PR antagonist, can enhance the response to gemcitabine chemotherapy [55]. S1PR1 is expected to be a druggable target that can improve ovarian cancer chemotherapy by promoting ovarian cancer cell senescence.

In summary, our experiments demonstrate that S1PR1 is involved in regulating ovarian cancer cell senescence through the S1PR1-PDK1-LATS1/2-YAP positive feedback signaling pathway. The schematic diagram of the molecular mechanism (Fig. 6J) shows that, when S1PR1 is activated by S1P or a related agonist in ovarian cancer cells, PDK1 expression is increased, LATS1/2 expression is inhibited, and YAP expression is increased, thereby inhibiting ovarian cancer cell senescence. YAP can further regulate the expression of S1PR1, and this positive feedback regulation leads to tumor cell senescence inhibition and exacerbated tumor cell proliferation. When the expression or activity of S1PR1 is inhibited, PDK1 expression is decreased and LATS1/1 is activated, leading to YAP inactivation and tumor cell senescence. Our study therefore revealed a potential target to treat ovarian cancer by inducing senescence. S1PR1 inhibition increases the sensitivity of ovarian cancer cells to CDDP by inducing senescence and may also provide a novel strategy to treat chemotherapy-resistant ovarian cancer.

## Materials and methods

### Experimental procedures

References to supplemental tables and figures are indicated with a prefix ‘S.’

#### Cell culture and CRISPR/Cas9 gene editing

A2780, ES-2, A2780-S, A2780-CP, SKOV3, OVCAR-3, and IOSE cells were cultured in DMEM (Gibco | Thermo Fisher Scientific, Waltham, MA, USA) containing 10% FBS (Gibco | Thermo Fisher Scientific) and 1% penicillin-streptomycin (Gibco | Thermo Fisher Scientific) at 37 °C and 5% CO_2_ (standard culture conditions). Granular cells (GC) were cultured in DMEM/F-12 (Gibco | Thermo Fisher Scientific) containing 10% FBS and 1% penicillin-streptomycin under standard culture conditions. HO8910 and C13 cells were cultured in RPMI 1640 (Gibco | Thermo Fisher Scientific) containing 10% FBS and 1% penicillin-streptomycin under standard culture conditions. S1PR1 knockout cells were constructed by CRISPR/Cas9 gene editing. Plasmids were transfected into A2780 and ES-2 cells by lentivirus-mediated transfection. Monoclonal cells were selected by 2 µg/mL puromycin screening for 3 days after 24 hours of transfection. S1PR1 knockout cells were selected by Western blotting to detect S1PR1 expression in the cells. S1PR1 sgRNA sequences are detailed in Table S1.1.

#### Cell proliferation and migration assay

Experimental methods are detailed in supporting information sections 4.1-4.4.

#### PI staining for cell cycle detection

Experimental methods are detailed in supporting information 4.5.

#### Total RNA extraction and quantitative PCR

Total RNA of ovarian cancer cells was extracted with TRIzol reagent (Invitrogen, Carlsbad, CA, USA). RNA concentration was measured by nucleic acid quantitative analyzer. The reverse transcription process was as follows: 65 °C for 5 min, rapid cooling on ice, 98 °C for 10 s, 55 °C for 15 s, 72 °C for 60 s, 30 cycles. The cDNA was diluted with DEPC water at a ratio of 1:5 and the control cDNA and experimental cDNA were detected by real-time quantitative PCR. qPCR analysis was performed using a SYBR Green master mix (Takara Bio, Kusatsu, Shiga, Japan) and a real-time PCR system (model 7300, Applied Biosystems | Thermo Fisher Scientific). The relative transcript levels were normalized to the mean transcript levels of the control group. Experiments were performed in triplicate. β-actin was used as the reference gene and the formula ΔΔCt was used to calculate mRNA levels. The primers are presented in Table S1.2.

#### Cell senescence assay

A total of 1 × 10^5^ control cells and S1PR1 knockout cells were counted, collected, and cultured overnight in 12-well plates. After washing with 1× PBS, 1× fixative solution was added and the cells were fixed at room temperature for 15 minutes, washed with 1 × PBS, and stained with senescence reagent (β-Galactosidase Staining Kit, Beyotime Biotechnology Co.) according to the manufacturer’s instructions. Stained cells were incubated overnight at 37 °C, washed with 1 × PBS, and imaged with a light microscope.

#### Immunohistochemical (IHC) analysis

Paraffin sections were dewaxed, rehydrated, and treated with 3% H_2_O_2_. Antigen restoration was performed with 10 μM sodium citrate (pH = 6.0). After 1 hour at room temperature, primary antibodies were incubated overnight at 4 °C. After the second antibody incubation, sections were washed with PBS (3 times, 5 minutes each time), and the secondary antibodies were incubated at room temperature for 30 minutes. Next, sections were colored with Vectastain ABC kit and 3,3 ‘-diaminobenzidine (DAB) peroxidase substrate kit (Vector Laboratories, Burlingame, CA, USA) and observed under a microscope. The antibodies are presented in Table S2.1.

#### Immunofluorescence analysis

The cells were cultured in 24-well plates and placed on slides for overnight culture. Then the cells were washed with PBS and fixed with 4% paraformaldehyde at room temperature for 30 minutes. The cells were then sealed with PBST containing 5% BSA. The primary antibody was incubated overnight at 4 °C (in the negative control group, PBS was used instead of primary antibody). Secondary antibodies were added and the cells were incubated at room temperature for 1 hour, stained with DAPI, and washed with PBS three times. Laser scanning confocal microscopy was used to acquire digital images (Olympus, Tokyo, Japan). The antibodies are described in Table S2.1.

#### Small interfering RNA (siRNA) transfection

A total of 1 × 10^5^ cells were seeded in each well of the 6-wells plate and the cells were cultured overnight. On the next day, 200 µL of serum-free medium, 8 µL of siRNA, and 8 µL of siRNA-MATE were added to a 1.5-mL Eppendorf tube and placed at room temperature for 15 minutes. The mixture was added to the cells and incubated at standard culture conditions for 36 hours. Cells were collected and assayed by fluorescence quantitative PCR and Western blotting. siRNA was synthesized by Gemma Genetics (Suzhou, China). The siRNA sequences are presented in Table S1.3.

#### Western blot analysis

The control cells and S1PR1 knockout cells were cultured overnight and counted. A total of 3 × 10^5^ control cells and S1PR1 knockout cells were collected in a 1.5-mL centrifuge tube, centrifuged at 12,000 r/min for 2 min, and the culture medium was discarded. The cells were resuspended in 100 μL of RIPA protein lysate to extract total protein. After protein concentration was determined with the BCA assay (Beyotime Biotechnology Co.), protein loading buffer was mixed evenly and placed in a metal heating block, heated at 95 °C for 5 minutes, and the protein was removed and snap frozen until further analysis. The protein sample was added to the concentrated gel through a microsyringe needle. The protein was transferred to PVD membrane (Millipore, Bedford, MA, USA) after a 90-minute electrophoresis run. Next, 5% skim milk powder was added and the gel was kept at room temperature for 1 hour and washed twice with 1 × TBST. The membrane was subsequently incubated with antibodies at 4 °C overnight. The antibodies used are described in Table S2.2.

After incubation, the membrane was removed and washed with TBST. The membrane was then incubated with the secondary antibody at room temperature for 1 hour (the secondary antibody was added to the 5% skim milk powder solution at a ratio of 1:5000) and the membrane was washed with TBST. After hybridization, ECL immunoblotting chemiluminescence kit was used for development.

#### Chromatin immunoprecipitation (ChIP)

ES-2 wild-type and S1PR1-deleted cells were fixed in 1% formaldehyde for 10 minutes and quenched with 0.125 M glycine for 5 minutes at room temperature (18 °C). Harvested cells were washed with ice-cold PBS and then lysed in ice-cold PBS containing protease inhibitor. The mixture was centrifuged at 2,000 × g for 5 minutes at 4 °C to pellet the nuclei, and the chromatin DNA was digested with micrococcal nuclease (MNase) in ChIP MNase buffer. Immunoprecipitation reactions were carried out with chromatin extracts overnight at 4 °C using 2 μg of antibodies against YAP. Histone H3 was used as the positive control and rabbit IgG (Cell Signaling Technology, Danvers, MA, USA) was used as the negative control. Two percent of the chromatin extract was set aside for input. Antibody-protein-DNA complexes were bound to 30 μL of ChIP-grade protein G magnetic beads (Cell Signaling Technology) for 2 hours at 4 °C. The beads were washed 3 times with low-salt wash buffer and once with high-salt wash buffer. Beads were resuspended in elution buffer and incubated for 30 minutes at 65 °C with frequent shaking. The resulting eluate and input samples were transferred into new tubes and reverse cross-linked by adding 6 μL of 5 M NaCl and 2 μL of proteinase K, and incubated for 2 hours at 65 °C. The DNA was purified using DNA spin columns. Precipitated DNA was quantitated by real-time quantitative PCR (RT-PCR) according to the manufacturer’s instructions (Simple ChIP Enzymatic Chromatin IP Kit, Cell Signaling Technology). The primers are presented in Table S1.4.

#### In vivo study

Sixteen SPF female nude mice (6-8 weeks old) were randomly divided into 2 groups (*n* = 8/group). In the control group, 5 × 10^5^ control ES-2 cells were injected subcutaneously into the dorsal flank of the animal under sterile conditions. In the S1PR1-null group, 5 × 10^5^ S1PR1-deleted ES-2 cells were injected into the dorsal flank of each animal. After 1 week, tumor size was measured using a Vernier caliper. When the tumor diameter was ≥15 mm, the animals were sacrificed (human end point). Subcutaneous tumors were removed for measurement.

#### Statistics

All in vitro experiments were repeated three times. Experimental data were statistically analyzed in Prism software (GraphPad Software, San Diego, CA, USA). A non-parametric test was used to compare the data between the experimental groups. A *p*-value of ≤ 0.05 was considered statistically significant. Significance levels are indicated as: *, *p* < 0.05; **, *p* < 0.01; ***, *p* < 0.001; and ns, *p* > 0.05.

### Supplementary information

supplemental Figure1

supplemental Figure2

Supplementary Materials

## Data Availability

All data described in these experiments are available upon request to the corresponding author.
