# Supplementary material for: S1PR1 regulates ovarian cancer cell senescence through the PDK1-LATS1/2-YAP pathway
Source: Oncogene. 2023 Oct 12;42(47):3491–502. doi: 10.1038/s41388-023-02853-w (PMC10656284; doi:10.1038/s41388-023-02853-w)
Supplement: Supplementary file 3 — Supplementary Materials [file 41388_2023_2853_MOESM3_ESM.docx]

**SUPPLEMENTAL MATERIAL**

# S1PR1 regulates ovarian cancer cell senescence through the PDK1-LATS1/2-YAP pathway

# Running title: S1PR1 and ovarian cancer cell senescence

Yi-Ping Tao^1,^^13#^, Heng-Yan Zhu^1,13#^, Qian-Yuan Shi^1,13#^, Cai-Xia Wang^1^, Yu-Xin Hua^1,2^, Han-Yin Hu^1,2^, Qi-Yin Zhou^1,2^, Zi-Lu Zhou^1^, Ying Sun^1^, Xiao-Min Wang^1^, Yu Wang^1^, Ya-Ling Zhang^1^, Yan-Jun Guo^1^, Zi-Ying Wang^1^, Xuan Che^3^, Chun-Wei Xu^4^, Xian-Chao Zhang^5^, Michal Heger^6,7,8^, Su-Ping Tao^9^, Xin Zheng^9^, Ying Xu^1^, Lei Ao^1^,Ai-Jun Liu^10^, Sheng-Bing Liu^1^*, Shu-Qun Cheng^11^*, Wei-Wei Pan^1,12^*

*^1^ Department of Cell Biology, College of Medicine, Jiaxing University, 118 Jiahang Road, Jiaxing 314001, China*

*^2^ Zhejiang Chinese Medicine University and Jiaxing University Master Degree Cultivation Base, Jiaxing University, 118 Jiahang Road, Jiaxing 314001, China*

*^3^ Department of Anesthesiology, Jiaxing Maternity and Child Health Care Hospital, Affiliated Women and Children Hospital, Jiaxing University, Zhejiang Province, China. Jiaxing 314001, China*

*^4^ Institute of Basic Medicine and Cancer (IBMC), Chinese Academy of Sciences,* *No. 1 Banshan East Street, Gongshu District,* *Hangzhou 310022,* *China*

*^5^* *Institute of Information Network and Artificial Intelligence, Jiaxing University, 118 Jiahang Road, Jiaxing 314001, China*

*^6^Jiaxing Key Laboratory for Photonanomedicine and Experimental Therapeutics, Department of Pharmaceutics, College of Medicine, Jiaxing University, 118 Jiahang Road, Jiaxing 314001, China*

*^7^ Department of Pharmaceutics, Utrecht Institute for Pharmaceutical Sciences, Utrecht University, Universiteitsweg 99, 3584 CG Utrecht, the Netherlands*

*^8^Laboratory of Experimental Oncology, Department of Pathology, Erasmus MC, Dr. Molewaterplein 40, 3015 GD Rotterdam, the Netherlands*

*^9^[Department of Gynecology and Obstetrics](http://www.baidu.com/link?url=MDNDMPnViAdb15U0e_OhEdWyz-DJGOJbtJemejip-OhWqCj8uYWUW1lg_W_eO6pBrLvUn_NgPDgLdNgvNAEGn1I3-9WgCPFpC6YXGNGA8vRM1lHvi42P1hauo6sP4YuL972utG98Zf0rrU7ybWOEEPA_gBdPdTuMP_dVHwH46rm), Affiliated Hospital of Jiaxing University, Jiaxing, 314000, China*

*^10^ Department of Pathology, the 7th Medical Center, General Hospital of PLA, Beijing 100700, China*

*^11^Department of Hepatic Surgery VI, Eastern Hepatobiliary Surgery Hospital, Second Military Medical University, 225 Changhai Road, Shanghai 200438, China*

*^12^ G60 STI Valley Industry & Innovation Institute, Jiaxing University, 118 Jiahang Road, Jiaxing 314001, China*

^13#^*These authors contributed equally: Yi-Ping Tao, Heng-Yan Zhu, Qian-Yuan Shi*

***Correspondence:**

Sheng-Bing Liu, College of Medicine, Jiaxing University, Jiaxing 314001, China. Tel: +86-573-83643850; E-mail: ycfbing@163.com, https://orcid.org/0000-0003-3751-4024

Shu-Qun Cheng, Eastern Hepatobiliary Surgery Hospital, Second Military Medical University, No. 225 Changhai Road, Shanghai 200433, China. Tel: +86-21-81875251; E-mail: chengshuqun@aliyun.com, https://orcid.org/0000-0001-6760-7470

Wei-Wei Pan, College of Medicine, Jiaxing University, Jiaxing 314001, China. Tel: +86-573-85624294; E-mail: wwpan@mail.zjxu.edu.cn, https://orcid.org/0000-0002-3574-3758

# Supporting information

**1. Figures S1 to S2**

**Supplemental figure legends**

**Filename: Sup-1(Fig S1 A-D)**

**Fig S1A.** Relative band density of P21, P27, P62 IGFBP7, PAI-1, histone H3, and p-H2AX protein expression in wild-type cells and S1PR1 knockout cells (A2780 and ES-2). GAPDH was used as control. Student’s *t*-test; ****p* < 0.001.

**Fig S1B.** Relative band density of LATS1/2, PDK1, YAP, and p-YAP protein expression after S1P treatment in ES-2 cells. GAPDH was used as control. One-way ANOVA; ns, *p* > 0.05, ***p* < 0.01****p* < 0.001.

**Fig S1C.** Relative band density of LATS1/2, PDK1, p-PDK1, YAP, and p-YAP protein expression in wild-type ovarian cancer cells and S1PR1-deficient ovarian cancer cells (A2780 and ES-2). GAPDH was used as control. Student’s *t*-test; **p* < 0.05, ****p* < 0.001.

**Fig S1D.** Relative band density of LATS1/2, PDK1, p-PDK1, IGFBP7, and PAI-1 protein expression in ES-2 cells after treatment with PDK1 inhibitor BX517. GAPDH was used as control. Student’s *t*-test; ****p* < 0.001.

Values represent mean ± SD of three independent experiments.

**Filename: Sup-2(Fig S2 A-G)**

**Figure S2A.** β-galactosidase staining was used to qualitatively (left) and quantitatively (right) analyze the senescence changes of A2780 cells after treatment with S1PR1 inhibitor W146. One-way ANOVA; ***p* < 0.01, ****p* < 0.001.

**Figure S2B.** q-PCR analysis of *YAP*, *LATS1*, *MST1*, *Ankrd1*, and *Cyr61* expression in wild-type and S1PR1 knockout cells (ES-2 cells and A2780 cells). Student’s *t*-test; **p* < 0.05, ***p* < 0.01, ****p* < 0.001.

**Figure S2C.** Western blot showing expression levels of LATS1, LATS2 and p-YAP after S1P treatment in A2780 cells. GAPDH was used as control. One-way ANOVA; ns, *p* > 0.05, ****p* < 0.001.

**Figure S2D.** Western blot showing the expression of S1PR1, PDK1, and LATS1 in ES-2 cells after S1P1 receptor antagonist W146 treatment. GAPDH was used as control. Student’s *t*-test; **p* < 0.05, ***p* < 0.01, ****p* < 0.001.

**Figure S2E.** q-PCR analysis of *P21* and *P53* after S1P and BX517 treatment of ovarian cancer cells. One-way ANOVA; **p* < 0.05, ***p* < 0.01, ****p* < 0.001.

**Figure S2F.** q-PCR analysis of *P53* expression in wild-type ES-2 cells and S1PR1 knockout ES-2 cells after CDDP treatment (0.125 µg/mL). One-way ANOVA; **p* < 0.05, ***p* < 0.01, ****p* < 0.001.

**Figure S2G.** β-galactosidase staining results showing the senescence of wild-type, S1PR1-knockout, and S1PR1-knockout cells with LATS1/2 silencing in A2780 cells. *Scale bar, 50 µm.* One-way ANOVA; ***p* < 0.01, ****p* < 0.001.

**Figure S2H.** β-galactosidase staining results showing the senescence of wild-type, S1PR1-knockout, and P21-knockdown A2780 cells. *Scale bar, 50µm.* One-way ANOVA; ****p* < 0.001.

Values represent mean ± SD of three independent experiments.

**2. Table S1. Relevant nucleic acid sequences involved in this study.**

Table S1.1. The S1PR1 sgRNA sequence used for CRISPR/Cas9 gene editing.

S1PR1 guid RNA-1: GAATTCCATGCCGGCGATGA

S1PR1 guid RNA-2: GGCGAGGAGACTGAACACGG

Table S1.2. The primers sequences (Sangon Biotech, Shanghai, China) used in real-time RT-PCR analysis.

| Name | Sequence |
| --- | --- |

Lats1: 5’-TCCTCCACCACCTCTCAACACTTC-3’ (forward)

5’-TGCCAACAGGAACAGAACTAATGCC-3’ (reverse)

Lats2: 5’-GACCCGAGGAATGAGCAGATTGTG-3’ (forward)

5’-GCTGGTGGTAGGACGCAAACG-3’ (reverse)

Ankrd1: 5’-CACTTCTAGCCCAVVVTGTGA-3’(forward)

5’-CCACAGGTTCCGTAATGATTT-3’ (reverse)

Ctgf: 5’-AGCTGACCTGGAGGAAAACA-3’ (forward)

5’-GACAGGCTTGGCGATTTTAG-3’ (reverse)

Cyr61: 5’-GCTCAGTCAGAAGGCAGACC-3’(forward)

5’-GTTCTTGGGGACACAGAGGA-3’(reverse)

P53: 5’-ACAACGTTCTGTCCCCCTTG-3’ (forward)

5’-CTGGCATTCTGGGAGCTTCA-3’ (reverse)

Mdm2: 5’-CCTGGCTCTGTGTGTAATAAG-3’ (forward)

5’-ATCCAACCAATCACCTGAATG-3’ (reverse)

MST1: 5’-GCGGGAAGGGTGAGGGCTAC-3’ (forward)

5’-ATCGGTGCTGATGCGGGATTTG-3’ (reverse)

GAPDH: 5’-GGAGCGAGATCCCTCCAAAAT-3’ (forward)

5’-GGCTGTTGTCATACTTCTCATGG-3’ (reverse)

P27: 5’-TTTAATTGGGTCTCAGGCAAAC-3’ (forward)

5’-CCCTTTTGTTTTGCGAAGAAGA-3’ (reverse)

P21: 5’-GATGGAACTTCGACTTTGTCAC-3’ (forward)

5’-GTCCACATGGTCTTCCTCTG-3’ (reverse)

Amotl2: 5’-AGGAGAAGAGTTGCCCACCTATGAG-3’ (forward)

5’-TCGAAGAGCTTCATCCTGTCGC-3’ (reverse)

Actin: 5’-ACAGTTGTTCTTCTGTGCGCCT-3’ (forward)

5’-CGTTCCAGCACTCTCCTGTT-3’ (reverse)

S1PR1: 5’-ATATCAGCGCGGACAAGGAG-3’ (forward)

5’-CTGCCAACAGGTCTGAGAGG-3’ (reverse)

Tabel S1.3. The siRNA sequences (GenePharma, Shanghai, China) used in RNA interference.

| Name | Sequence |
| --- | --- |

Negative control - sense: 5’-UUCUCCGAACGUGUCACGUTT-3’

Negative control - antisense: 5’-ACGUGACACGUUCGGAGAATT-3’

siLATS1-1 - sense: 5’-UAGCAUGGAUUUCAGUAAUTT-3’

siLATS1-1 - antisense: 5’-AUUACUGAAAUCCAUGCUATT-3’

siLATS1-2 - sense: 5’-GGUGAAGUCUGUCUAGCAATT-3’

siLATS1-2 - antisense: 5’-UUGCUAGACAGACUUCACCTT-3’

siLATS2-1 - sense: 5’-GCACGCAUUUUACGCCUUATT-3’

siLATS2-1 - antisense: 5’-UAAGGCGUAAAAUGCGUGCTT-3’

siLATS2-2 - sense: 5’-ACACUCACCUCGCCCAAUATT-3’

siLATS2-2 - antisense: 5’-UAUUGGGCGAGGUGAGUGUTT-3’

siP21- sense: 5’- AACGGUGGAACUUCGACUUUGTT-3’

siP21- antisense: 5’-CAAAGUCGAAGUUCCACCGUUTT-3’

Table S1.4. The primers sequences (Sangon Biotech) used in chromatin immunoprecipitation (ChIP).

| Name | Sequence |
| --- | --- |

S1PR1 F GTCTTAGAGGACCGTTATCTTG

S1PR1 R AGACACACCCTGTACCTGAAT

**3. Table S2. Description of experimentally relevant antibodies and reagents**

**Table S2.1. Immunohistochemical (IHC) analysis and immunofluorescence analysis relevant antibodies.**

S1PR1 (Affinity Biosciences, Cincinnati, OH, USA, #DF4867, #DF2785), p-histone H3 (Cell Signaling Technology, Danvers, MA, USA, #9701), LATS1 (Cell Signaling Technology, #3477), LATS2 (Cell Signaling Technology, #5888), YAP (Santa Cruz Biotechnology, #SC-101199, Santa Cruz, CA, USA), P62 (Affinity Biosciences, #AF5384), and cleaved caspase-3 (Cell Signaling Technology, #9661).

**Table S2.2. Western blot analysis relevant antibodies.**

GAPDH (Bioke, Hangzhou, China, # BK7021), LATS1 (Cell Signaling Technology, #3477), LATS2 (Cell Signaling Technology, #5888), S1PR1 (Affinity Biosciences, #DF2785), PDK1 (Cell Signaling Technology, #3062), p-PDK1 (Cell Signaling Technology, #3061), YAP (Santa Cruz Biotechnology, Santa Cruz, CA, USA, #SC-101199), p-YAP (Cell Signaling Technology), P21 (Cell Signaling Technology), P27 (Cell Signaling Technology,#3686), P62 (Affinity Biosciences, #AF5384) cyclin B1 (Cell Signaling Technology, #4138), p-H2AX (Cell Signaling Technology), lamin B1 (Proteintech Group, Rosemont, IL, USA, #12987-1-AP), histone H3 (Proteintech Group, #17168-1-AP), MST1 (Cell Signaling Technology), IGFBP7 (Proteintech Group, #19961-1-AP), PAI-1 (Proteintech Group, # 66261-1-Ig)

**Table S2.3. S1P, W146, BX517, and cisplatin (CDDP).**

S1P (MedChemExpress, Monmouth Junction, NJ, USA, #HY-108496), W146 (R&D Systems, Minneapolis, MN, USA, #3602), BX517 (MedChemExpress, #HY-13842), and cisplatin (CDDP) (MedChemExpress, #HY-17394).

**4. Related experimental methods**

**4.1** | **Cell proliferation assay**

A total of 1 × 10^5^ control cells and the same number of S1PR1-knockout cells (A2780 / ES-2) were seeded in 6-well plates overnight. The number of cells in each well was counted at 24, 48, and 72 hours for 3 consecutive days, and a cell proliferation curve was constructed. The cells were seeded in 96-well plates at 5,000 cells per well, and cell viability was determined using the Cell Counting Kit-8 (MedChemExpress).

**4.2** | **Cell scratch assay**

S1PR1 knockout ovarian cancer cells and control cells were seeded into 24-well plates at 1 × 10^5^ cells/well, and within 24 hours, the cells formed monolayers. A straight line was engraved in the monolayer with the front end of a pipette. Next, the cells were washed twice with PBS to remove the detached cells. Fresh serum-free medium was added to the cells. Images were taken at the specified time points, the area was measured at different specified time points, and the wound closure rate was calculated.

**4.3** | **Transwell migration**

Cell culture medium containing 10% FBS was placed in the lower chamber. Serum-free medium was added to the top of the transwell membrane and then seeded with ovarian cancer cells, followed by incubation at standard culture conditions for 24 hours. Migrating/invading cells at the bottom of the membrane were fixed with 4% paraformaldehyde, stained with 0.5% crystal violet for 15 min, and counted.

**4.4** | **Soft agar colony formation assay**

The lower layer was comprised of 0.5% agar. A 1.5-mL mixture was transferred into a 6-wells plate and left to stand at room temperature for 15 minutes until the gel solidified. Control cells and S1PR1-knockout cells were prepared into a single cell suspension, mixed with 0.35% agar top gel, and transferred into 6-wells plates at 2,500 cells per well. After standing for 15 minutes at room temperature, 2 mL of DMEM was added to the solidified agar. DMEM culture medium was replaced at an interval of 2 days, and the culture was maintained at standard culture conditions for 14-20 days. The number of clones (more than 50 cells/colony) was calculated after staining with crystal violet.

**4.5** | **PI staining for cell cycle detection**

Control cells and S1PR1-knockout cells were cultured overnight and counted. A total of 1 × 10^6^ cells was collected in a 1.5-mL centrifuge tube, centrifuged at 800 r/min for 6 min, and the supernatant was discarded. Cells were washed with 1 mL of pre-cooled 1× PBS. The cells were resuspended in 300 μL of precooled 1 × PBS and slowly added to a 1.5-mL centrifuge tube containing 700 μL precooled anhydrous ethanol and fixed overnight. On the second day, 1 mL of fixative solution containing cells was transferred to a 15-mL centrifuge tube, 2 mL of precooled 1 × PBST was added, and the cells were centrifuged at 1000 r/min for 8 min. The supernatant was decanted and the cell cycle was measured by flow cytometry.
